# Understanding confidence in the human papillomavirus vaccine in Japan: a web-based survey of mothers, female adolescents, and healthcare professionals

**DOI:** 10.1080/21645515.2021.1918042

**Published:** 2021-06-01

**Authors:** Michiko Shuto, Youngju Kim, Kotoba Okuyama, Kazunobu Ouchi, Hideo Ueichi, Chimeremma Nnadi, Heidi J. Larson, Gonzalo Perez, Shin Sasaki

**Affiliations:** a Medical Affairs, MSD K.K., Kitanomaru Square, Chiyoda, Tokyo, Japan; b Department of Pediatrics, Kawasaki Medical School, Kurashiki, Japan; c Faculty of Engineering, Information and Systems, University of Tsukuba, Tsukuba, Japan; d Merck Sharp & Dohme Corp., A Subsidiary of Merck & Co., Inc., Kenilworth, NJ, USA; e Department of Infectious Disease Epidemiology, LSHTM, London, UK; f Department of Health Metrics Sciences, University of Washington, Seattle, WA, USA; g Department of Clinical Research, Universidad del Rosario, Sede Quinta Mutis, Bogotá, Colombia

**Keywords:** Cervical cancer, cross-sectional study, HPV, HPV vaccine, Japan, vaccine confidence

## Abstract

Vaccine confidence reflects social, individual, and political factors indicating confidence in vaccines and associated health systems. In Japan, the government ceased proactive recommendation of the human papillomavirus (HPV) vaccine in June 2013, only several months after the recommendation had begun. Seven years later, as of October 2020, the suspension persists and vaccine coverage has precipitously declined, resulting in many young women being continually exposed to the risk of preventable HPV-related diseases. Accordingly, understanding stakeholder opinions on HPV vaccination issues is critical for informing strategies to improve HPV vaccine confidence and acceptance. In October 2019, we performed a nationwide, web-based survey of 1646 mothers of HPV-vaccination–eligible girls, 562 female adolescents aged 15–19 years, and 919 healthcare professionals (HCPs) in Japan. This survey captured key elements of vaccine confidence (i.e., importance, effectiveness, and safety of the HPV vaccine), awareness, and the willingness to receive (in HPV-vaccination–eligible girls) or recommend (in HCPs) the HPV vaccine, and the factors responsible for these decisions. HPV vaccine confidence was generally higher among HCPs than among mothers or female adolescents. Nearly half of all stakeholders were neutral regarding their willingness to receive/recommend the HPV vaccine. The seriousness of cervical cancer and the HPV vaccine’s effectiveness or safety were important deciding factors for receiving/recommending the HPV vaccine. Besides these factors, sufficient information and free vaccination were crucial. Our results suggest several factors that could help shape public policy and communication strategies to improve HPV vaccine confidence and acceptance in Japan.

## Introduction

In Japan, in 2018, cervical cancer was the second most common cancer among women aged 15–44 years.^1^ Human papillomaviruses (HPVs) cause almost all cases of cervical cancer and are implicated in various other cancers.^1^ Many countries have invested significant resources in HPV vaccination and other measures to promote cervical cancer elimination.^2^ In Japan, HPV vaccines have been commercially available since late 2009 and were introduced into the national immunization program (NIP) in April 2013.

Robust evidence supports the effectiveness and safety of HPV vaccines.^3–7^ Nonetheless, media reports about the occurrence of diverse symptoms in some HPV vaccine recipients in 2013 led the Ministry of Health, Labour and Welfare (MHLW) in Japan to suspend their proactive recommendation for routine use of the HPV vaccine in the NIP just a few months after their recommendation began. The suspension persists. HPV vaccine coverage has sharply declined from approximately 70% to less than 1%.^8^ Among Japanese women born between 1994 and 2007, an excess of 24,600–27,300 preventable cervical cancer cases and 5000–5700 cervical cancer deaths are projected to occur over their lifetime owing to the precipitous decline in HPV vaccination coverage recorded between 2013 and 2019.^9^

Vaccine confidence is defined as trust in the importance, effectiveness, and safety of vaccines and the health system in which those are approved and delivered.^10,11^ Understanding and implementing effective approaches to restoring confidence in the HPV vaccine is critical to improving and sustaining vaccination acceptance to potentially avert adverse public health consequences.^10^ We therefore conducted a survey focused on understanding HPV vaccine confidence and the willingness to receive the HPV vaccine among Japanese mothers and female adolescents, and among healthcare professionals (HCPs) to recommend the HPV vaccine.

## Methods

### Study design

This was a nationwide, cross-sectional, web-based survey of mothers of HPV vaccination–eligible girls, female adolescents, and HCPs in Japan.

### Study populations

Mothers and female adolescents were recruited from a database managed by INTAGE Healthcare, Inc. (Tokyo, Japan). HCPs were recruited from a database managed by PLAMED, Inc. (Tokyo, Japan). Inclusion criteria included mothers who have at least one daughter aged 12–16 years, female adolescents aged 15–19 years, and HCPs who are gynecologists (obstetricians/gynecologists), pediatricians, or internists who had seen a female adolescent aged 12–19 years in clinical practice within the year prior to study participation. Although the recommended age for HPV vaccination in Japan starts at 12 years, girls aged 12–14 years were excluded because of the respondent panel’s age restriction. Outcomes were assessed among (i) mothers who have daughters unvaccinated against HPV, (ii) female adolescents who have never received the HPV vaccine, and (iii) HCPs who currently do not recommend HPV vaccines to their patients aged 12–16 years. Factors associated with receiving or recommending the HPV vaccine were also assessed among mothers who have daughters vaccinated against HPV, female adolescents who have received the HPV vaccine, and HCPs who currently recommend HPV vaccines to their patients aged 12–16 years.

### Outcomes

The primary outcome was measures of HPV vaccine confidence (i.e., perceived importance, effectiveness, and safety of the HPV vaccine) and awareness of cervical cancer and the HPV vaccine among mothers, female adolescents, and HCPs. Secondary outcomes included HPV vaccination practices and the willingness to receive, have their daughters receive (for mothers), or (for HCPs) recommend the HPV vaccine, and factors associated with receiving or recommending the HPV vaccine, including timing and factors associated with acceptance of the HPV vaccine.

### Study procedure and assessments

Potentially eligible respondents were randomly sampled and sent e-mail invitations with a web-link to participate in the study. A maximum of three reminders were sent to survey participants. An eligibility check was performed. The web-link included in the e-mail invitation directed participants to a webpage providing the following: (i) relevant study information, (ii) the option to provide informed consent for study participation and data utilization for possible publication, and (iii) screening questions (i.e., approximately five items). Only participants who provided informed consent and met the inclusion/exclusion criteria were able to proceed and respond to the web-based study questionnaire. Respondents were allowed to respond to the questionnaire only once. Mothers with more than one daughter were requested to answer questions only for the daughter whose age was closest to 13 years. All respondents were asked basic demographic questions. The total number of items per respondent was approximately 30. The survey questionnaires were developed based on items in the Vaccine Confidence Index™ developed by the Vaccine Confidence Project as a tool for estimating vaccine confidence, against a mix of socio-demographic variables, and using a five-point Likert scale: Strongly agree, Tend to agree, Do not know, Tend to disagree, and Strongly disagree, for three domains of confidence.^10,11^ Other questions for this study were developed based on previous studies and modified for the situation in Japan.^12,13^ Question wording was finalized after a pilot study conducted from August 28 to 30, 2019, to ensure question accuracy and comprehensibility.

To prevent missing or out-of-range numerical values, restrictions were implemented on the online response entries. Upon completing the entire questionnaire, respondents received points that could be exchanged for various items, such as cash and gift cards.

Vaccine confidence was defined as a composite of answers to questions about the HPV vaccine, assessing vaccine importance, effectiveness, and safety. Other questions assessed levels of vaccine confidence regarding views on the importance, effectiveness, and safety of non–HPV vaccines, trust in the government to protect citizens from health risks, awareness of cervical cancer and the HPV vaccine, and factors associated with vaccination/recommendation. For mothers and adolescents, adolescents’ HPV vaccination history was queried, as was HCPs’ current status for HPV vaccine recommendation.

### Ethics approval

Respondents’ consent for study participation and data utilization was obtained electronically by having them click a checkbox. The study protocol was approved by the ethics committee of Kitamachi Clinic (Tokyo, Japan; approval number: QVD06742), and the study was conducted in accordance with the Ethical Guidelines for Medical and Health Research Involving Human Subjects and the Act on the Protection of Personal Information in Japan. Under this act, surveys were anonymized to prevent personal information from being traceable by the sponsor to identify a specific individual.

### Statistical analysis

The analysis was primarily descriptive in nature; thus, no formal hypothesis tests were conducted. For continuous variables, descriptive statistics (i.e., mean, standard deviation) were calculated. For categorical variables, the number and proportion of respondents within each corresponding category were calculated. All statistical analyses were performed using SAS version 9.4 (SAS Institute) and R version 3.6.2 (R Core Team, 2019). The proportions for each outcome were summarized descriptively in subgroups by each covariate. Pearson’s chi-square test was used to analyze statistical differences in the proportions for any comparisons between groups, with a *P*-value of <0.05 considered as statistically significant.

## Results

**Figure 1.**
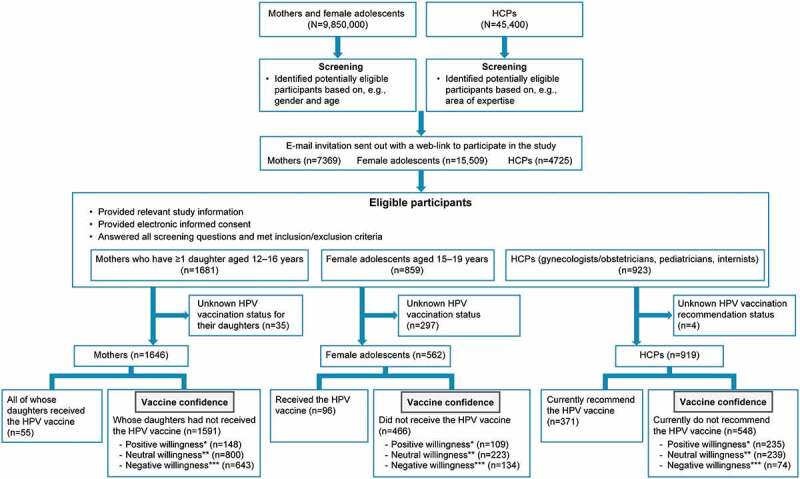
Study flow diagram. Mothers who have ≥1 daughter aged 12–16 years and female adolescents aged 16–19 years were randomly sampled given the large number of such individuals registered in the database, but e-mail invitations were sent to all female adolescents aged 15 years who were registered in the database. Gynecologists/obstetricians, pediatricians, and internists were randomly sampled

The survey was conducted in October 2019, with 1646 mothers, 562 female adolescents, and 919 HCPs included in the analysis populations. We describe vaccine confidence, awareness, and willingness to receive/recommend the HPV vaccine among 1591 mothers with daughters unvaccinated against HPV, 466 female adolescents who had never received the HPV vaccine, and 548 HCPs who at the time of the survey did not recommend the HPV vaccine to their patients. For all respondents, the factors that determined their decisions were assessed (Figure 1). Tables 1–3 show the respondents’ demographic and background information.

**Table 1.** Demographic and baseline characterstics of mothers (n=1646)

	n (%)
Age of mothers, years	
Mean±SD	45.5±4.8
Age of daughters, years	
12	318 (19)
13	353 (21)
14	379 (23)
15	371 (23)
16	384 (23)
Geographic residence	
Hokkaido	92 (6)
Tohoku	66 (4)
Kanto	591 (36)
Chubu	241 (15)
Kinki	354 (22)
Chugoku	105 (6)
Shikoku	41 (3)
Kyushu	156 (10)
Highest education level	
Middle school	29 (2)
High school	477 (29)
Short training or college degree	669 (41)
University	415 (25)
Graduate school and above	32 (2)
Other than the above	4 (0)
Do not want to respond	20 (1)
Employment status	
Employed	773 (47.0)
Self-employed or enterprise manager	51 (3.1)
Full-time homemaker	691 (42.0)
Student	2 (0.1)
Other than the above	129 (7.8)

SD = standard deviation.

**Table 2.** Demographic and baseline characterstics of female adolescents (n=562)

	n (%)
Age of female adolescents, years	
Mean±SD	17.1±1.1
15	2 (0)
16	130 (23)
17	131 (23)
18	131 (23)
19	168 (30)
Geographic residence	
Hokkaido	27 (5)
Tohoku	36 (6)
Kanto	174 (31)
Chubu	112 (20)
Kinki	114 (20)
Chugoku	20 (4)
Shikoku	17 (3)
Kyushu	62 (11)
Current educational level or employment status	
Middle school	3 (1)
High school	364 (65)
Short training or college degree	30 (5)
University	117 (21)
Employed	12 (2)
Self-employed or enterprise manager	0 (0)
Full-time homemaker	6 (1)
Other than the above	30 (5)

SD = standard deviation.

**Table 3.** Demographic and baseline characterstics of HCPs (n=919)

	n (%)
Age of HCPs, years	
20–29	12 (1)
30–39	139 (15)
40–49	243 (26)
50–59	304 (33)
60–69	192 (21)
≥70	29 (3)
Gender	
Male	810 (88)
Geographic location of medical practice	
Hokkaido	48 (5)
Tohoku	42 (5)
Kanto	302 (33)
Chubu	152 (17)
Kinki	198 (22)
Chugoku	58 (6)
Shikoku	26 (3)
Kyushu	93 (10)
Number of patients given HPV vaccines	
0	316 (34)
1–19	249 (27)
20–99	277 (30)
≥100	77 (8)
Vaccination history of patients	
Do you recommend any vaccines, including HPV vaccines?	
Yes, and consequently most patients get vaccinated	576 (63)
Yes, but most patients do not get vaccinated	148 (16)
No, but most patients get vaccinated	87 (10)
No, and most patients do not get vaccinated	108 (12)

HCP = healthcare professional; HPV = human papillomavirus.

*As reflected by answers of “Willing” or “Very willing.” **As reflected by an answer of “Do not know.” ***As reflected by answers of “Not willing” or “Very unwilling.”HCP = healthcare professional; HPV = human papillomavirus.

### Vaccine confidence

**Figure 2.**
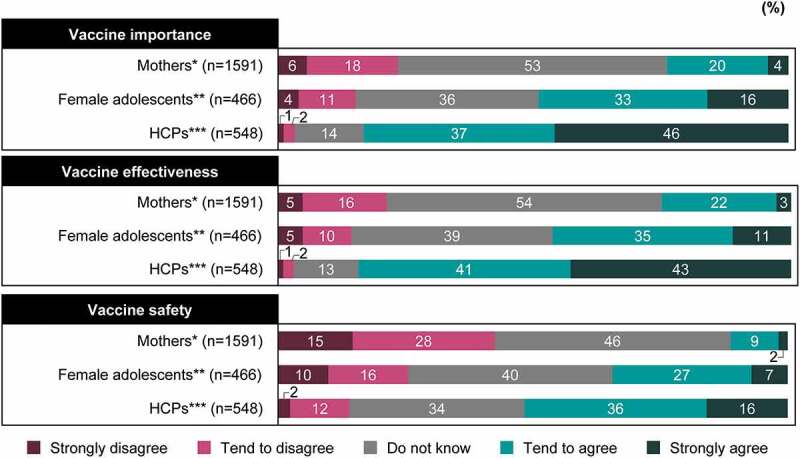
HPV vaccine confidence. HPV vaccine importance: “Overall, I think HPV vaccines are important to have”; HPV vaccine effectiveness: “Overall, I think HPV vaccines are effective”; HPV vaccine safety: “Overall, I think HPV vaccines are effective.” *Mothers who have daughters unvaccinated against HPV. **Female adolescents who never received the HPV vaccine. ***HCPs who do not currently recommend HPV vaccination to their patients. *P*<.001 for between-group comparison for the proportions of “Tend to agree” and “Strongly agree.”

Positive endorsement (i.e., “Tend to agree” or “Strongly agree”) on the importance, effectiveness, and safety of the HPV vaccine was reported by 24%, 25%, and 11% of mothers; 49%, 46%, and 34% of female adolescents; and 83%, 84%, and 52% of HCPs, respectively (*P*< .001). Mothers (46%–54%) and female adolescents (36%–40%) most commonly selected “Do not know” for questions related to the three factors of vaccine confidence (Figure 2).

HCP = healthcare professional; HPV = human papillomavirus.

Levels of HPV vaccine confidence were higher among female adolescents in the Chugoku region than in other regions (Supplementary Figure 1). Views on vaccines and vaccination in infants/toddlers and adolescents are shown in Supplementary Figure 2. The levels of confidence in non–HPV vaccines were higher than those in the HPV vaccine.

Ten percent of mothers and 18% each of female adolescents and HCPs agreed with the statement, “The information about HPV vaccines from the government is sufficient” (Supplementary Figure 3).

### Awareness

**Figure 3.**
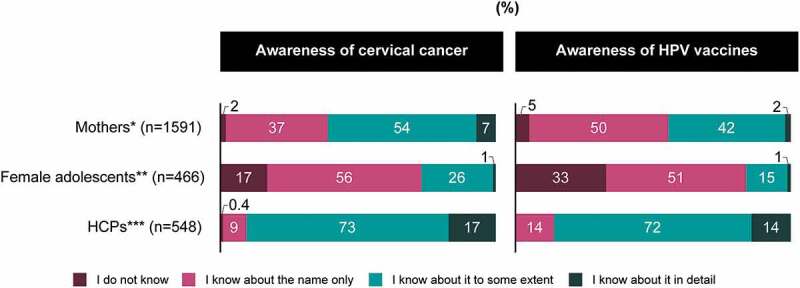
Awareness of cervical cancer and the HPV vaccine. *Mothers who have daughters unvaccinated against HPV. **Female adolescents who never received the HPV vaccine. ***HCPs who do not currently recommend HPV vaccination to their patients. *P*<.001 for between-group comparison for the proportions of “I know about it to some extent” and “I know about it in detail for both items.”

Positive response (i.e., “I know about it to some extent” or “I know about it in detail”) to the awareness of cervical cancer and the HPV vaccine was reported by 61% and 44% of mothers, 27% and 16% of female adolescents, and 90% and 86% of HCPs, respectively (*P*<.001 for both items). Approximately one-third of female adolescents were not aware of the HPV vaccine (Figure 3). Female adolescents who knew about HPV vaccines in detail or to some extent had low confidence in HPV vaccine importance (Supplementary Figure 4). Levels of confidence in HPV vaccine effectiveness and safety exhibited similar trends (data not shown). There were no large differences among female adolescents regarding the information source influencing these trends (data not shown). However, more female adolescents with higher awareness had low confidence in HPV vaccine importance and thought, “HPV vaccine recommendations are currently revoked because of potential safety issues” than female adolescents with high confidence in HPV vaccine importance (data not shown).

HCP = healthcare professional; HPV = human papillomavirus.

Among HCPs, there were differences across medical specialization regarding awareness of the HPV vaccine. Sixty-seven percent of general practitioner (GP) gynecologists and 71% of hospital physician (HP) gynecologists—more than twice the proportion of any other specialists—answered that they knew about the HPV vaccine in detail. Eleven percent of HP internists, 58% of GP pediatricians, and 32%–55% of other specialists knew that the HPV vaccine is included in Japan’s NIP.

### Willingness to receive or recommend the HPV vaccine

**Figure 4.**
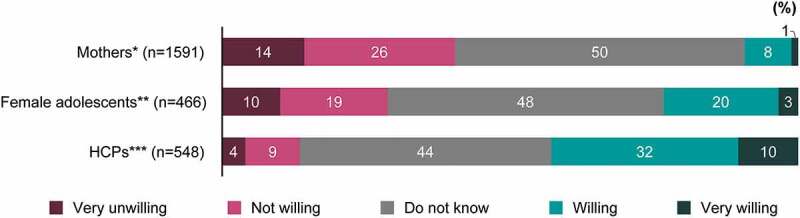
Willingness to receive or recommend the HPV vaccine. *Mothers who have daughters unvaccinated against HPV. **Female adolescents who never received the HPV vaccine. ***HCPs who do not currently recommend HPV vaccination to their patients. *P*<.001 for between-group comparison for the proportions of “Willing” to receive or recommend the HPV vaccine

Among mothers and female adolescents, 9% and 23%, respectively, were willing to receive the HPV vaccine, whereas 43% of HCPs were willing to recommend the HPV vaccine (*P*<.001). However, neutral willingness (answer “Do not know”) was the largest proportion in all groups (Figure 4). Respondents with higher HPV vaccine confidence were more willing to receive or recommend the HPV vaccine (Supplementary Figure 5).

HCP = healthcare professional; HPV = human papillomavirus.

**Figure 5.**
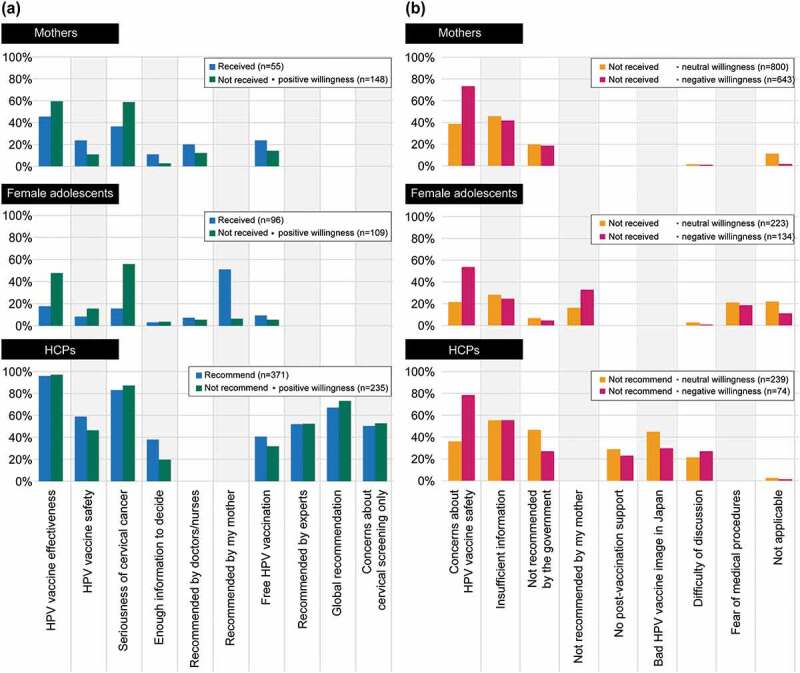
(a) Reasons for having received or to recommend the HPV vaccine (multiple answers allowed), and (b) reasons for not to decide to have received or not to decide to recommend the HPV vaccine (multiple answers allowed). Main reasons present only those items that had a response rate of 20% or more in at least one of the three groups. Please refer to supplementary tables for further details on all items

HCP = healthcare professional; HPV = human papillomavirus.

### Reasons and sources of information for the decision to receive/recommend the HPV vaccine

The main reasons why mothers chose to have their daughters vaccinated were because they thought HPV vaccines are effective (45%) and cervical cancer is dangerous (36%; Supplementary Table 1). The mother’s recommendation was the main reason for female adolescents having received the HPV vaccine (Figure 5(a); Supplementary Table 2). At the time of the survey, 40% of HCPs recommended HPV vaccination to their female adolescent patients. HCPs stated that they currently recommend HPV vaccination because of its effectiveness (96%), the seriousness of cervical cancer (83%), and their belief that HPV vaccines are safe (59%; Figure 5(a); Supplementary Table 3). “Safety concerns” and “insufficient information” were the main drivers among the stakeholders for vaccine refusal or lack of recommendation (Figure 5(b); Supplementary Tables 4–6).

**Figure 6.**
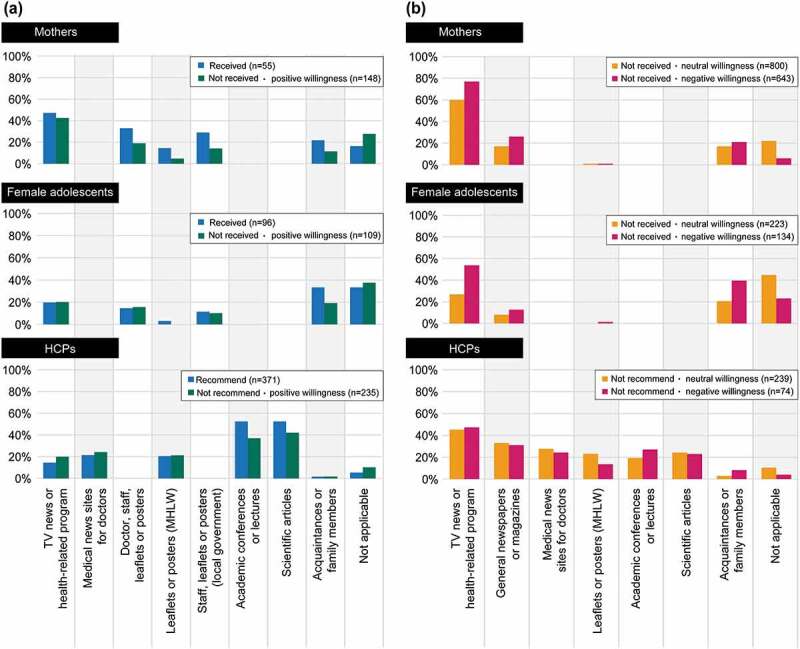
Sources of information used to decide whether (a) or not (b) to receive or recommend the HPV vaccine (multiple answers allowed). Main sources of information present only those items that had a response rate of 20% or more in at least one of the three groups. Please refer to supplementary tables for further details on all items

Figure 6 shows sources of information used by mothers and female adolescents to decide whether or not to get vaccinated against HPV or, for HCPs, whether to recommend HPV vaccination. HCPs who selected television (TV) news or health-related programs as their primary source of information were less willing to recommend the HPV vaccine. Among sources of information (multiple answers possible) used to decide whether or not to receive the HPV vaccine, TV news or health-related programs were used by 43%–77% of mothers and 20%–54% of female adolescents; social networking services or video-sharing sites were indicated by 3%–5% in both groups (Supplementary Table 7; see also Supplementary Table 8).

HCP = healthcare professional; HPV = human papillomavirus; MHLW = Ministry of Health, Labour and Welfare; TV = television.

**Figure 7.**
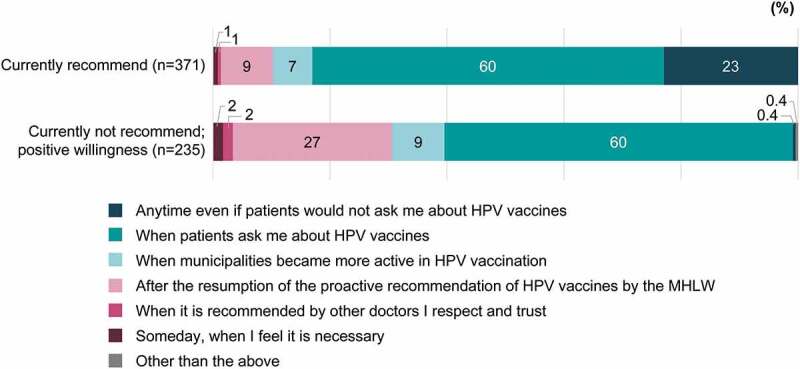
Timing of recommendation for HCPs who currently recommend or who are willing to recommend the HPV vaccine (in response to the question, “When would you like to recommend HPV vaccines to your patients?”). When the HCPs who recommend HPV vaccines “Anytime even if patients would not ask me about HPV vaccines” and those who recommend “When patients ask me about the vaccines” were asked their reasons, there were differences of more than 15 points between these two groups with regard to “Safety (I think HPV vaccines are safe),” “Sufficient information (We know enough about HPV vaccines),” and “Routine vaccination (HPV vaccines are free for girls of an HPV vaccine–eligible age).”

**Figure 8.**
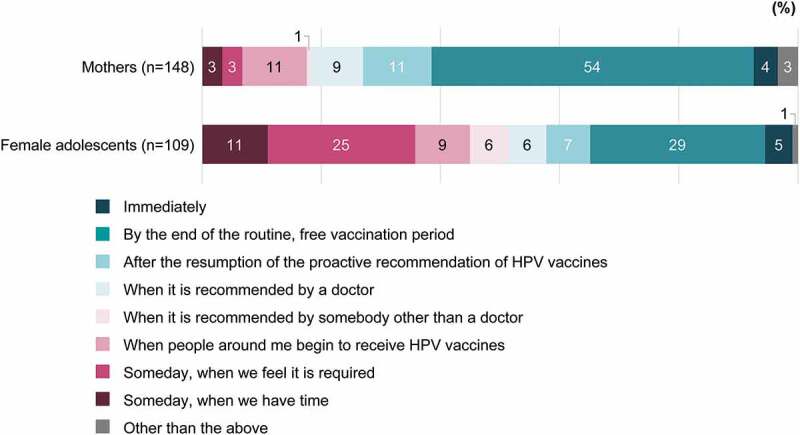
Timing of HPV vaccination for mothers who are willing to have their daughters receive the HPV vaccine (when asked, “When would you like to have your daughter(s) receive HPV vaccines?”) and female adolescents who are willing to receive the HPV vaccine (when asked, “When would you like to receive the HPV vaccine?”)

Sixty percent each of HCPs who currently recommend the HPV vaccine and HCPs who do not but stated their willingness to do so reported that they would recommend the HPV vaccine based on patient inquiry (Figure 7). Supplementary Tables 9 and 10 show sources of information used by HCPs to decide whether or not to recommend the HPV vaccine. Almost 60% of the mothers willing to have their daughters receive the HPV vaccine and approximately one-third of the female adolescents expressed a positive opinion (i.e., endorsement of “Immediately” or “By the end of the routine, free vaccination period”) regarding the timing of vaccination (Figure 8).

HCP = healthcare professional; HPV = human papillomavirus; MHLW = Ministry of Health, Labour and Welfare.

HPV = human papillomavirus.

## Discussion

This is the first nationwide, comprehensive survey on vaccine confidence related to HPV vaccination in Japan conducted since the 2013 suspension of the government’s proactive HPV vaccine recommendation. Collectively, the findings could help inform the development of effective public health communications, public engagement, and advocacy campaigns that could lead to greater awareness of, confidence in, and improved uptake of the HPV vaccine in Japan.

Key findings are that HPV vaccine confidence and willingness to receive/recommend the HPV vaccine were higher among HCPs than among mothers and female adolescents (*P*<.001). Among mothers, HPV vaccine confidence regarding safety was slightly lower than that for effectiveness and importance. Overall, respondents with high HPV vaccine confidence were more willing to receive/recommend the HPV vaccine. The seriousness of cervical cancer, the HPV vaccine’s effectiveness and safety, sufficient information, and free HPV vaccination were important factors for respondents’ decisions to receive/recommend the HPV vaccine. Sixty percent of HCPs who currently recommend the HPV vaccine or who were willing to do so each reported that they would recommend it based on patient inquiry. As patients rarely ask their physicians about the HPV vaccine, this result indicates that HCPs are not necessarily recommending the HPV vaccine proactively or that often.

With regard to the timing of HPV vaccination, the results suggest that female adolescents’ intention to receive the HPV vaccine was lower than that of mothers to have their daughters receive the vaccine. Overall, results indicate female adolescents as being less eager for HPV vaccination, although this could also reflect their lack of awareness and possibly their relative inaction compared with their mothers.

The most common reason for neutral willingness of vaccination among mothers and female adolescents was “insufficient information.” In fact, approximately one-third of the female adolescents were not aware of the HPV vaccine. Moreover, with the current stand of the Japanese government to withhold resumption of proactive recommendation of the HPV vaccine in the NIP and HCPs not recommending the vaccine proactively, it could be expected that mothers and female adolescents were unable to decide on HPV vaccination.

Converging sources of evidence in Japan^4–7^ and globally^3^ support the high effectiveness of the HPV vaccine against HPV infection, cervical cancer, and related diseases. Our results thus underscore gross disparities between the perceptions of key stakeholders and actual vaccine risks. For example, the HPV vaccine has been shown to be safe and not causally associated with the adverse events publicized by the media.^14–16^ Nonetheless, proactive recommendation of the HPV vaccine by the MHLW remains suspended, despite recommendations by the Japan Expert Council on Promotion of Vaccination.^17^ Numerous other countries have devoted substantial resources to HPV vaccination and other strategies to facilitate cervical cancer elimination.^2^ Given the high stakes involved in Japan, swift political action has been urged.^18^

A 2018 internet-based public opinion survey administered throughout Japan to 1000 men and women aged >20 years reported that <30% of respondents had basic knowledge about the HPV vaccine and 67% could not decide whether or not HPV vaccination should be recommended.^19^ Among Japanese physicians, 90% indicated that they would restart vaccinating adolescents against HPV if the MHLW resumed their proactive recommendation of the vaccine.^20^ Similar results are supported by other studies,^21^ including regarding mothers’ perception of the HPV vaccine and their intentions to have their daughters vaccinated.^22,23^ Acceptance of the HPV vaccine was higher among parents working in healthcare, who were knowledgeable about the HPV vaccine and cervical cancer.^22^ Clear and unbiased information about HPV, cervical cancer, and the HPV vaccine, along with proactive recommendation by the MHLW, could thus be key determinants of parents’ and female adolescents’ willingness to accept HPV vaccination. This is especially true given that although trust in doctors and nurses in Japan is generally high, confidence in vaccines is low, underscoring wider issues relating to trust in the government and their policies.^24^

Previous surveys^19–23^ confirm the low levels of public trust, interest, and awareness of the HPV vaccine among parents, adolescents, and HCPs in Japan, although no nationwide data on HPV vaccine confidence and willingness with its timing have been published. Thus, investigating HPV vaccine confidence nationwide across Japan among the three important stakeholders—mothers, female adolescents, and HCPs—and identifying the factors that determine their willingness to receive or (for HCPs) recommend the HPV vaccine are crucial to better understand behavioral motives and the dynamics of the interaction and communication between them that could in turn be addressed by tailored interventions.

Public trust and confidence in the HPV vaccine and its providers, as well as in the vaccination process, development policies, and recommendation, are at the core of increasing vaccine acceptance and implementing successful immunization programs. Our results reveal the current state of HPV vaccine confidence in Japan and its possible determinants. Such evidence could help shape public policy and communication strategies to improve HPV vaccine confidence and acceptance in Japan.

We found that the level of awareness/understanding and type and source of information shape perception of the HPV vaccine. HCPs recommending the HPV vaccine or who were willing to do so relied on background scientific knowledge more than other sources of information. Moreover, HCPs who referred to scientific information showed high acceptance of the HPV vaccine. By contrast, HCPs, as well as mothers and female adolescents who were negative about the HPV vaccine, were more likely to be influenced by media reporting. Both mothers and female adolescents selected “TV news or health-related programs” as the most frequently used source of information. Mothers willing to recommend the HPV vaccine were influenced by information from the local government and HCPs.

A high proportion of the HCPs we surveyed were not even aware that the HPV vaccine is included in the NIP. Based on these results, better public communication is needed around the availability of the HPV vaccine in the NIP. HCPs who were asked about the reasons for recommending the HPV vaccine reported that safety, sufficient information, routine (free) vaccination, and care and support after vaccination were the most important.

Since mothers’ and female adolescents’ reliance on information about the safety and effectiveness of the HPV vaccine can influence willingness to receive the HPV vaccine, all stakeholders should be given sufficient and appropriate information about the HPV vaccine. Findings from this study could help shape public policy and communication strategies to improve future HPV vaccine confidence and acceptance in Japan and ultimately increase HPV vaccination rates to avert the rising trend of cervical cancer.^9^ Including the HPV vaccine along with routine adolescent vaccinations has been shown in the United States to be a key determinant of HPV vaccination rates.^25^ We found that many mothers, female adolescents, and HCPs have higher confidence in vaccines for infants and toddlers compared with the HPV vaccine.

Because of the differences between Japan and other countries regarding HPV vaccine policy, some of our results may not be widely generalizable. Within Japan, however, both INTAGE and PLAMED databases have similar patterns of distribution of age, gender, geographic locations, and clinical departments as nationwide data. The selected database populations are not expected to greatly differ from the target Japanese populations because the database from INTAGE Healthcare, Inc.^26^ (as of October 2018) consisted of approximately 9,850,000 individuals in Japan and that of PLAMED, Inc.^27^ (as of April 2016) consisted of approximately 45,400 doctors from 37 medical departments in Japan. The analysis population included 34 female adolescents who reported that they had received the HPV vaccine at the age of 9 years or 10 years—ages ineligible for the routine vaccination program in Japan. Results excluding these respondents were similar to those that included them (data not shown).

Several limitations should be noted. Respondents’ characteristics might be different from those of the general population because people without internet access were not included, nor were females aged 12–14 years. Mothers, females aged 16–19 years, and HCPs were randomly sampled given the large number of such individuals registered in the database. In contrast, all registered females aged 15 years received an e-mail invitation because of the relatively small number of such individuals in the database. Response bias may have occurred in this study as it is likely that individuals who responded to the questionnaire had an interest in the research area compared with those who did not, which could distort the samples’ representativeness. The causal relationships between factors associated with the intentions of the respondents cannot be determined because of the cross-sectional nature of this study. Additionally, the intended willingness to receive or recommend the HPV vaccine may not reflect actual behavior. An important clinical question revealed by the current study is what can bridge the gap between willingness and behavior.

In conclusion, levels of HPV vaccine confidence were higher among HCPs than among mothers and female adolescents. The seriousness of cervical cancer and perceptions of HPV vaccine effectiveness and safety were the most important factors among all stakeholders for the decision to receive/recommend the HPV vaccine. Mothers and female adolescents were more likely to be influenced by media reporting, but some of them indicated the desire to get information from HCPs. Given that patients rarely ask their physicians about the HPV vaccine, this represents an impediment to HCPs recommending the HPV vaccine in the future. This is because HCPs stated that their willingness to recommend the HPV vaccine would be based on patient inquiry. This thus represents another gap that could be addressed through future interventions. Overall, our survey results suggest the existence of several critical factors that could change the perception and behavior around HPV vaccination. These in particular include resumption of a proactive recommendation by the MHLW and HCP recommendation.

## Supplementary Material

Supplemental Material
