# Supplementary material for: Understanding confidence in the human papillomavirus vaccine in Japan: a web-based survey of mothers, female adolescents, and healthcare professionals
Source: Hum Vaccin Immunother. 2021 Jun 1;17(9):3102–12. doi: 10.1080/21645515.2021.1918042 (PMC8577382; doi:10.1080/21645515.2021.1918042)
Supplement: Supplemental Material [file KHVI_A_1918042_SM7051.docx]

**Supplementary Data**

**Understanding confidence in the human papillomavirus vaccine in Japan: a web-based survey of mothers, female adolescents, and healthcare professionals**

Michiko Shuto, Youngju Kim, Kotoba Okuyama, Kazunobu Ouchi, Hideo Ueichi, Chimeremma Nnadi, Heidi J. Larson, Gonzalo Perez, and Shin Sasaki

# Supplementary Figure 1. Vaccine confidence by region for (A) mothers, (B) female adolescents, and (C) HCPs


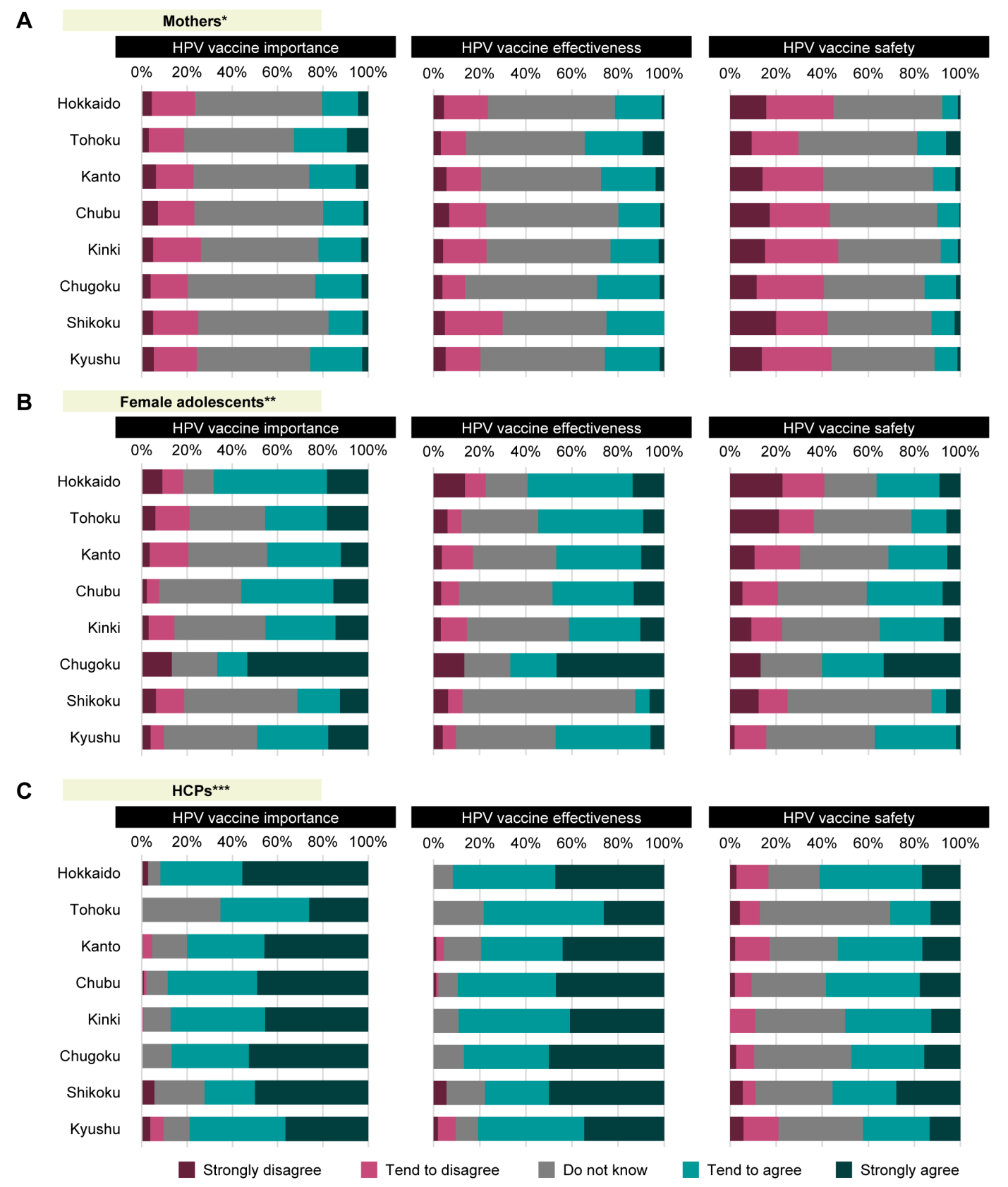


*Mothers who have daughters unvaccinated against HPV. **Female adolescents who never received the HPV vaccine. ***HCPs who do not currently recommend HPV vaccination to their patients.

HCP = healthcare professional; HPV = human papillomavirus.

**Supplementary Figure 2. Views on vaccine (A) importance, (B) effectiveness, and (C) safety**


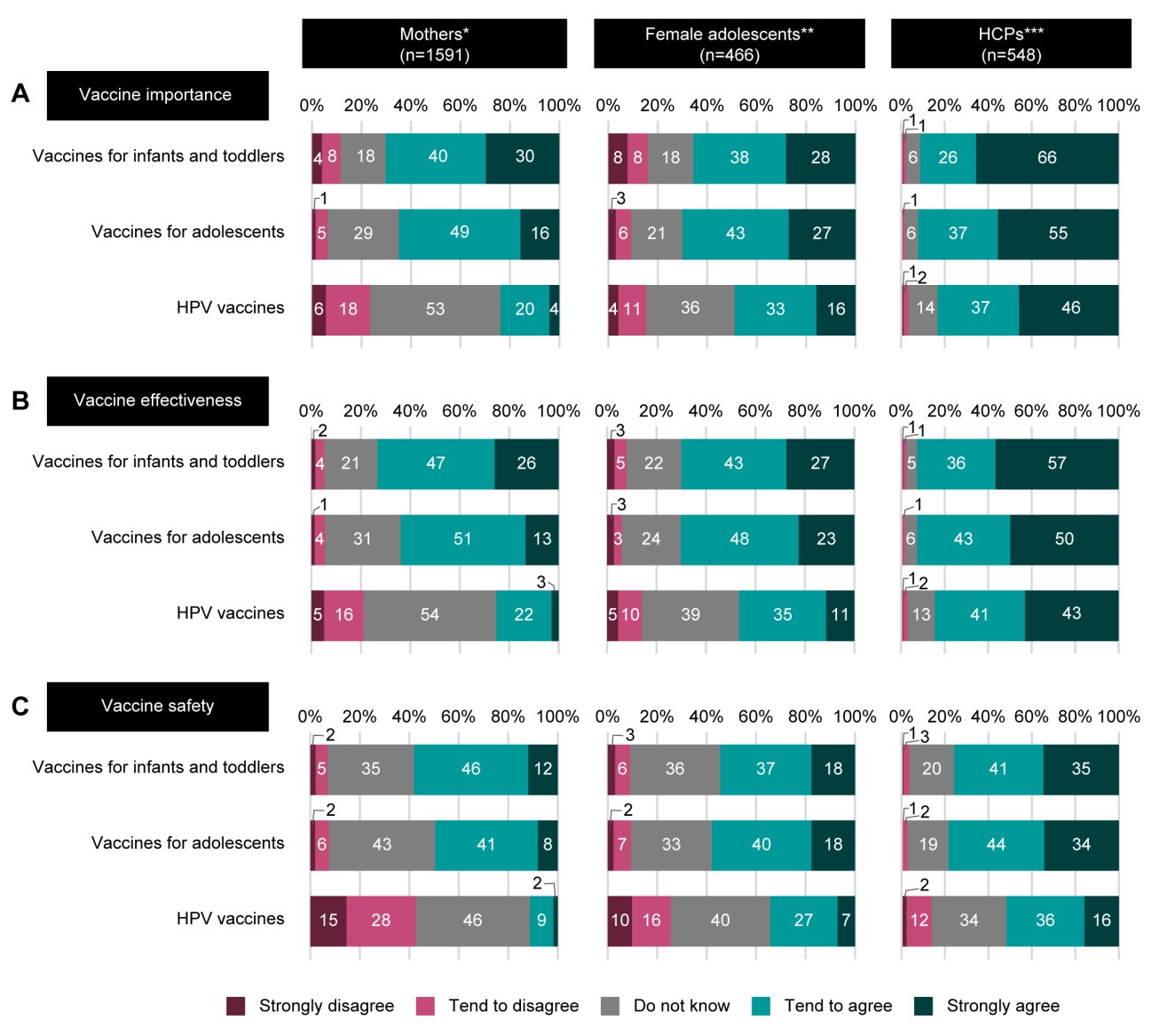


*Mothers who have daughters unvaccinated against HPV. **Female adolescents who never received the HPV vaccine. ***HCPs who do not currently recommend HPV vaccination to their patients.

HCP = healthcare professional; HPV = human papillomavirus.

**Supplementary Figure 3. Views on the government regarding health and HPV vaccine information**


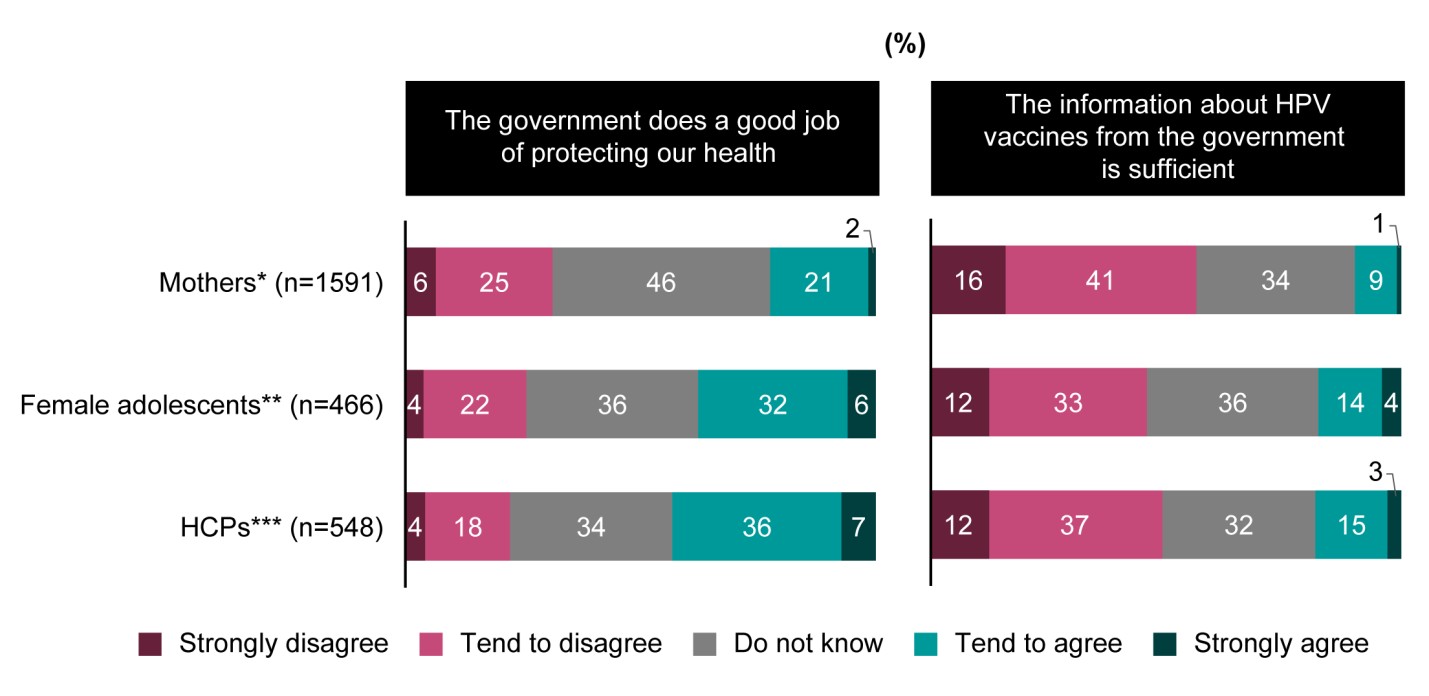


*Mothers who have daughters unvaccinated against HPV. **Female adolescents who never received the HPV vaccine. ***HCPs who do not currently recommend HPV vaccination to their patients.

HCP = healthcare professional; HPV = human papillomavirus.

**Supplementary Figure 4. Levels of confidence in HPV vaccine importance based on awareness of the HPV vaccine**


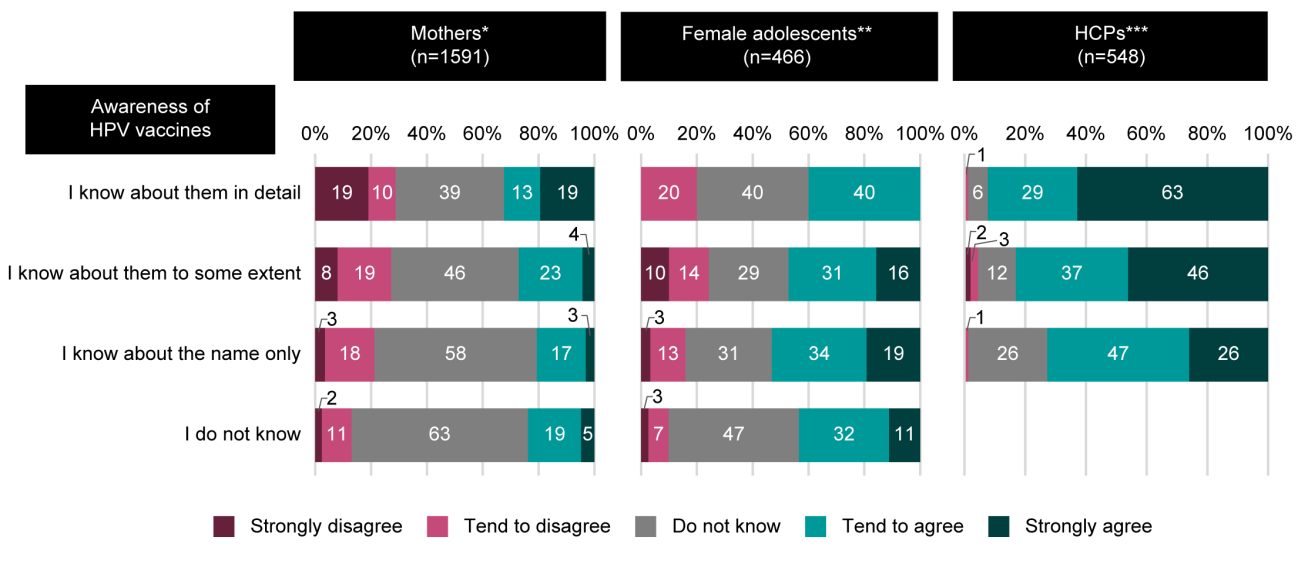


*Mothers who have daughters unvaccinated against HPV. **Female adolescents who never received the HPV vaccine. ***HCPs who do not currently recommend HPV vaccination to their patients.

HCP = healthcare professional; HPV = human papillomavirus.

**Supplementary Figure 5. (A) Mothers’* HPV vaccine confidence based on their willingness for their daughters to receive the HPV vaccine; (B) female adolescents’** HPV vaccine confidence based on their willingness to receive the HPV vaccine; (C) HCPs’*** HPV vaccine confidence based on their willingness to recommend the HPV vaccine**


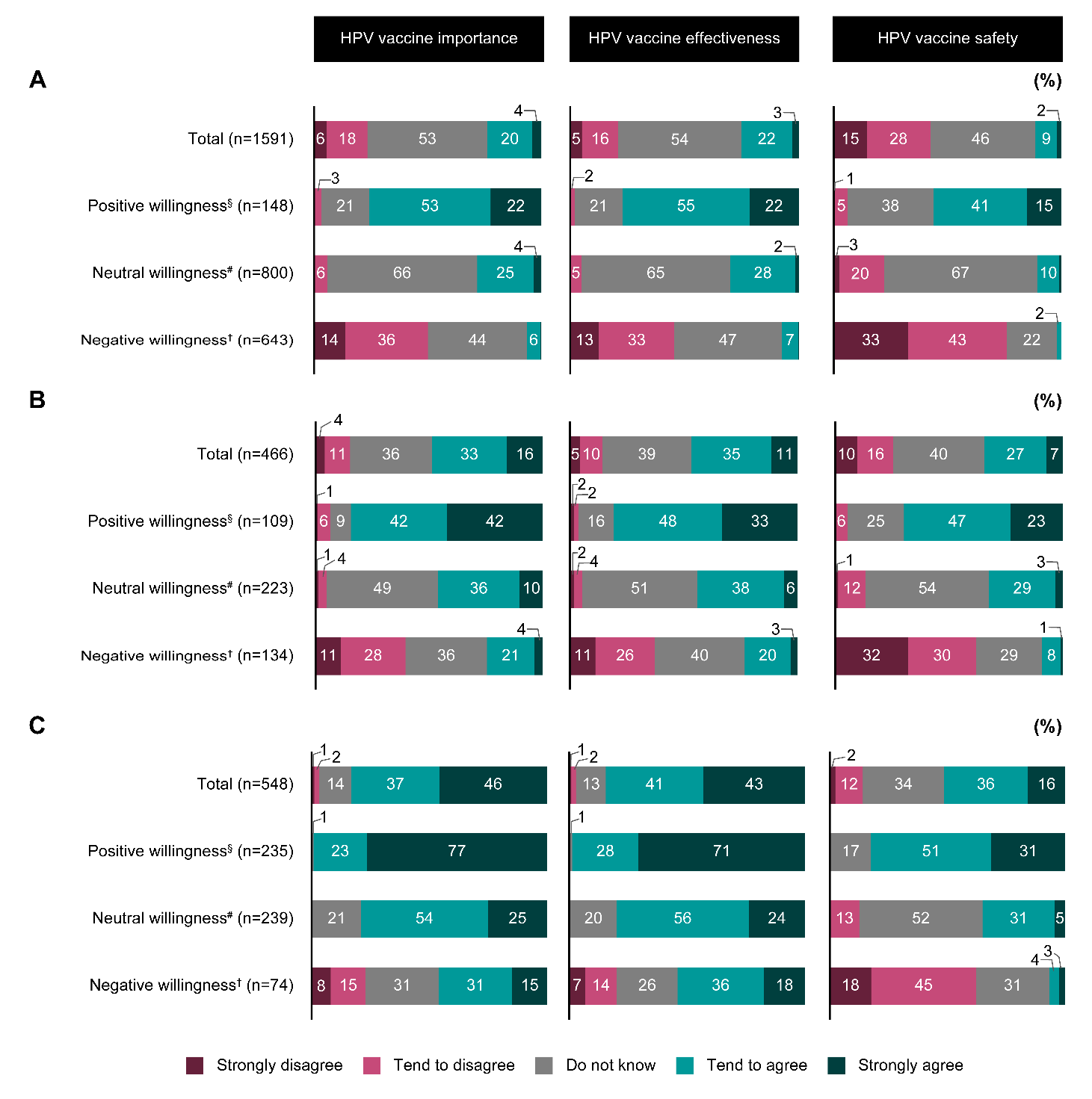


*Mothers who have daughters unvaccinated against HPV. **Female adolescents who never received the HPV vaccine. ***HCPs who do not currently recommend HPV vaccination to their patients. ^§^As reflected by answers of “Willing” or “Very willing.” ^#^As reflected by an answer of “Do not know.” ^†^As reflected by answers of “Not willing” or “Very unwilling.”

HCP = healthcare professional; HPV = human papillomavirus.

**Supplementary Table 1. Reasons for mothers having their daughters receive the HPV vaccine or being willing for them to receive it (multiple answers allowed)**

|  | **Received (n=55)** | **Positive willingness* (n=148)** |
| --- | --- | --- |
| I think HPV vaccines are effective | 25 (45.45) | 88 (59.46) |
| I think HPV vaccines are safe | 13 (23.64) | 16 (10.81) |
| I think cervical cancer is dangerous | 20 (36.36) | 87 (58.78) |
| My friends’ daughters receive HPV vaccines | 6 (10.91) | 1 (0.68) |
| I have enough information to make a decision | 6 (10.91) | 4 (2.70) |
| I think it is possible that my daughter may get infected with HPV in the future | 7 (12.73) | 27 (18.24) |
| I think it is the right time for my daughter to get the HPV vaccination | 7 (12.73) | 15 (10.14) |
| I have a responsibility to my daughter to get her the HPV vaccination | 8 (14.55) | 12 (8.11) |
| The government recommends the HPV vaccination | 3 (5.45) | 12 (8.11) |
| Doctors or nurses recommend the HPV vaccination | 11 (20.00) | 18 (12.16) |
| My husband (partner) recommends the HPV vaccination | 2 (3.64) | 3 (2.03) |
| My family members (other than husband) recommend the HPV vaccination | 4 (7.27) | 1 (0.68) |
| School or teachers recommend the HPV vaccination | 3 (5.45) | 0 (0.00) |
| My daughter wants to get the HPV vaccination | 4 (7.27) | 0 (0.00) |
| I did not have any bad vaccination experiences in the past | 2 (3.64) | 2 (1.35) |
| HPV vaccines are free for my daughter now | 13 (23.64) | 21 (14.19) |
| Other than the above | 0 (0.00) | 2 (1.35) |
| Not applicable | 7 (12.73) | 11 (7.43) |

Data are reported as n (%). *As reflected by answers of “Willing” or “Very willing.”

HPV = human papillomavirus.

# Supplementary Table 2. Reasons for female adolescents having received the HPV vaccine or being willing to receive it (multiple answers allowed)

|  | **Received (n=96)** | **Positive willingness* (n=109)** |
| --- | --- | --- |
| I think HPV vaccines are effective | 17 (17.71) | 52 (47.71) |
| I think HPV vaccines are safe | 8 (8.33) | 17 (15.60) |
| I think cervical cancer is dangerous | 15 (15.63) | 61 (55.96) |
| My friends receive HPV vaccines | 6 (6.25) | 5 (4.59) |
| I have enough information to make a decision | 3 (3.13) | 4 (3.67) |
| I think it is possible that I may get infected with HPV in the future | 3 (3.13) | 6 (5.50) |
| I think it is a good time for me to receive the HPV vaccine | 6 (6.25) | 3 (2.75) |
| The government recommends HPV vaccination | 11 (11.46) | 8 (7.34) |
| Doctors or nurses recommend HPV vaccination | 7 (7.29) | 6 (5.50) |
| My mother recommends HPV vaccination | 49 (51.04) | 7 (6.42) |
| My father recommends HPV vaccination | 6 (6.25) | 1 (0.92) |
| My family members (other than mother or father) recommend HPV vaccination | 11 (11.46) | 8 (7.34) |
| My school or teachers recommend HPV vaccination | 10 (10.42) | 4 (3.67) |
| I do not have any concerns about any vaccinations | 2 (2.08) | 3 (2.75) |
| HPV vaccines are free for girls of an HPV‑vaccine–eligible age | 9 (9.38) | 6 (5.50) |
| Other than the above | 3 (3.13) | 3 (2.75) |
| Not applicable | 19 (19.79) | 10 (9.17) |

Data are reported as n (%). *As reflected by answers of “Willing” or “Very willing.”

HPV = human papillomavirus.

# Supplementary Table 3. Reasons for HCPs to currently recommend the HPV vaccine or to be willing to recommend it (multiple answers allowed)

|  | **Recommend (n=371)** | **Positive willingness* (n=235)** |
| --- | --- | --- |
| I think HPV vaccines are effective | 356 (95.96) | 228 (97.02) |
| I think HPV vaccines are safe | 219 (59.03) | 109 (46.38) |
| I think cervical cancer is dangerous | 308 (83.02) | 205 (87.23) |
| I got enough information to make a decision | 141 (38.01) | 46 (19.57) |
| The government recommends HPV vaccination | 45 (12.13) | 17 (7.23) |
| HPV vaccines are free for girls of an HPV‑vaccine–eligible age | 151 (40.70) | 75 (31.91) |
| Other doctors, experts, or academia recommend HPV vaccination | 193 (52.02) | 123 (52.34) |
| HPV vaccines are recommended globally | 249 (67.12) | 172 (73.19) |
| Cervical screening is not sufficient to prevent cervical cancer | 187 (50.40) | 124 (52.77) |
| Post–HPV vaccination supports have been prepared | 69 (18.60) | 20 (8.51) |
| My patients inquire about HPV vaccines | 45 (12.13) | 10 (4.26) |
| HPV vaccination is a revenue driver | 15 (4.04) | 5 (2.13) |
| Other than the above | 0 (0.00) | 0 (0.00) |
| Not applicable | 0 (0.00) | 1 (0.43) |

Data are reported as n (%). *As reflected by answers of “Willing” or “Very willing.”

HCP = healthcare professional; HPV = human papillomavirus.

# Supplementary Table 4. Reasons for mothers not to decide to have their daughters receive the HPV vaccine or being unwilling for them to receive it (multiple answers allowed)

|  | **Neutral willingness* (n=800)** | **Negative willingness****  **(n=643)** |
| --- | --- | --- |
| I think HPV vaccines are not effective or cannot prevent cervical cancer | 29 (3.63) | 57 (8.86) |
| I think HPV vaccines are not safe | 309 (38.63) | 472 (73.41) |
| I think cervical cancer is not dangerous | 4 (0.50) | 6 (0.93) |
| My friends’ daughters do not receive HPV vaccines | 73 (9.13) | 48 (7.47) |
| I do not get enough information to make a decision | 366 (45.75) | 268 (41.68) |
| I do not think my daughter will be infected with HPV in the future | 7 (0.88) | 4 (0.62) |
| I think it is too early or late for my daughter to get the HPV vaccine | 17 (2.13) | 12 (1.87) |
| I (or we) do not have enough time | 11 (1.38) | 6 (0.93) |
| I (or we) think it is too much bother to get HPV vaccination | 12 (1.50) | 2 (0.31) |
| The government does not recommend HPV vaccination | 157 (19.63) | 120 (18.66) |
| Doctors or nurses do not recommend HPV vaccination | 101 (12.63) | 75 (11.66) |
| My husband (partner) does not recommend HPV vaccination | 4 (0.50) | 5 (0.78) |
| My family members (other than husband) do not recommend HPV vaccination | 24 (3.00) | 22 (3.42) |
| School or teachers do not recommend HPV vaccination | 48 (6.00) | 26 (4.04) |
| My daughter does not want to get the HPV vaccine | 33 (4.13) | 34 (5.29) |
| I hesitate to have my daughter receive any vaccines | 28 (3.50) | 46 (7.15) |
| It is hard to discuss this with my daughter (or other family members) | 12 (1.50) | 6 (0.93) |
| HPV vaccines cost money | 68 (8.50) | 33 (5.13) |
| Other than the above | 55 (6.88) | 50 (7.78) |
| Not applicable | 89 (11.13) | 11 (1.71) |

Data are reported as n (%). *As reflected by an answer of “Do not know”. **As reflected by answers of “Not willing” or “Very unwilling.”

HPV = human papillomavirus.

# Supplementary Table 5. Reasons for female adolescents not to decide to have received the HPV vaccine or being unwilling to receive it (multiple answers allowed)

|  | **Neutral willingness* (n=223)** | **Negative willingness****  **(n=134)** |
| --- | --- | --- |
| I think HPV vaccines are not effective or cannot prevent cervical cancer | 13 (5.83) | 20 (14.93) |
| I think HPV vaccines are not safe | 48 (21.52) | 72 (53.73) |
| I think cervical cancer is not dangerous | 0 (0.00) | 0 (0.00) |
| My friends do not receive HPV vaccines | 20 (8.97) | 10 (7.46) |
| I do not get enough information to make a decision | 63 (28.25) | 33 (24.63) |
| I do not think that I will get infected with HPV in the future | 2 (0.90) | 4 (2.99) |
| I think it is too early or late for me to receive HPV vaccines | 5 (2.24) | 3 (2.24) |
| I do not have enough time | 12 (5.38) | 4 (2.99) |
| I think it is too much bother to get HPV vaccination | 17 (7.62) | 4 (2.99) |
| The government does not recommend HPV vaccination | 15 (6.73) | 6 (4.48) |
| Doctors or nurses do not recommend HPV vaccination | 20 (8.97) | 12 (8.96) |
| My mother does not recommend HPV vaccination | 36 (16.14) | 44 (32.84) |
| My father does not recommend HPV vaccination | 6 (2.69) | 9 (6.72) |
| My family members (other than mother or father) do not to recommend HPV vaccination | 18 (8.07) | 18 (13.43) |
| My school or teachers do not recommend HPV vaccination | 9 (4.04) | 3 (2.24) |
| It is hard to discuss this with my mother (or other family members) | 6 (2.69) | 1 (0.75) |
| HPV vaccines cost money | 32 (14.35) | 8 (5.97) |
| I fear medical procedures involving injections | 47 (21.08) | 25 (18.66) |
| Other than the above | 9 (4.04) | 6 (4.48) |
| Not applicable | 49 (21.97) | 15 (11.19) |

Data are reported as n (%). *As reflected by an answer of “Do not know”. **As reflected by answers of “Not willing” or “Very unwilling.”

HPV = human papillomavirus.

# Supplementary Table 6. Reasons for HCPs not to decide to recommend the HPV vaccine, or to be unwilling to recommend it (multiple answers allowed)

|  | **Neutral willingness* (n=239)** | **Negative willingness****  **(n=74)** |
| --- | --- | --- |
| I think HPV vaccines are not effective | 13 (5.44) | 6 (8.11) |
| I think HPV vaccines are not safe | 86 (35.98) | 58 (78.38) |
| I think cervical cancer is not dangerous | 6 (2.51) | 1 (1.35) |
| I do not get enough information to make a decision | 132 (55.23) | 41 (55.41) |
| The government does not actively recommend HPV vaccination | 111 (46.44) | 20 (27.03) |
| HPV vaccines are not included in the national immunization program | 27 (11.30) | 10 (13.51) |
| Other doctors, experts, or academia do not recommend HPV vaccination | 17 (7.11) | 6 (8.11) |
| HPV vaccines have many issues globally | 11 (4.60) | 6 (8.11) |
| Cervical screening is sufficient to prevent cervical cancer | 6 (2.51) | 6 (8.11) |
| Post–HPV vaccination supports have never been prepared | 69 (28.87) | 17 (22.97) |
| HPV vaccines have a bad image in Japan | 107 (44.77) | 22 (29.73) |
| It is hard to talk with HPV-vaccine–eligible young adolescents or their parents (or family members) | 51 (21.34) | 20 (27.03) |
| HPV vaccines cost money (including financial concerns such as keeping the vaccine in stock) | 7 (2.93) | 2 (2.70) |
| Other than the above | 7 (2.93) | 3 (4.05) |
| Not applicable | 6 (2.51) | 1 (1.35) |

Data are reported as n (%). *As reflected by an answer of “Do not know”. **As reflected by answers of “Not willing” or “Very unwilling.”

HCP = healthcare professional; HPV = human papillomavirus.

# Supplementary Table 7. Sources of information used to decide whether to receive the HPV vaccine (multiple answers allowed)

|  | **Mothers** | | **Female adolescents** | |
| --- | --- | --- | --- | --- |
|  | **Received (n=55)** | **Positive willingness* (n=148)** | **Received (n=96)** | **Positive willingness* (n=109)** |
| TV news or health-related programs | 26 (47.27) | 63 (42.57) | 19 (19.79) | 22 (20.18) |
| Newspapers or magazines | 10 (18.18) | 16 (10.81) | 11 (11.46) | 6 (5.50) |
| Internet (news website) | 2 (3.64) | 7 (4.73) | 5 (5.21) | 6 (5.50) |
| Internet (pharmaceutical company website or health-related information site) | 3 (5.45) | 4 (2.70) | 4 (4.17) | 1 (0.92) |
| Internet (academia or hospital/clinic website) | 8 (14.55) | 7 (4.73) | 3 (3.13) | 1 (0.92) |
| Internet (MHLW website) | 10 (18.18) | 4 (2.70) | 4 (4.17) | 2 (1.83) |
| Internet (local government) | 7 (12.73) | 5 (3.38) | 3 (3.13) | 1 (0.92) |
| Internet (SNS [SM] or video-sharing site) | 2 (3.64) | 5 (3.38) | 5 (5.21) | 3 (2.75) |
| Internet (websites other than the above) | 0 (0.00) | 2 (1.35) | 0 (0.00) | 0 (0.00) |
| Doctors, staff, leaflets, or posters (academia hospital/clinic) | 18 (32.73) | 28 (18.92) | 14 (14.58) | 17 (15.60) |
| Leaflets or posters (MHLW) | 8 (14.55) | 7 (4.73) | 3 (3.13) | 0 (0.00) |
| Staff, leaflets, or posters (local government or public health center) | 16 (29.09) | 21 (14.19) | 11 (11.46) | 11 (10.09) |
| Leaflets or posters (school) | 1 (1.82) | 0 (0.00) | 3 (3.13) | 0 (0.00) |
| Lessons (school) | NA | NA | 11 (11.46) | 10 (9.17) |
| Information from acquaintances or family members | 12 (21.82) | 17 (11.49) | 32 (33.33) | 21 (19.27) |
| Public lectures or seminars by experts | 7 (12.73) | 5 (3.38) | 6 (6.25) | 6 (5.50) |
| Other than the above | 2 (3.64) | 7 (4.73) | 4 (4.17) | 5 (4.59) |
| Not applicable | 9 (16.36) | 41 (27.70) | 32 (33.33) | 41 (37.61) |

Data are reported as n (%). *As reflected by answers of “Willing” or “Very willing.”

HPV = human papillomavirus; MHLW = Ministry of Health, Labour and Welfare; NA = not applicable; SM = social media; SNS = social networking services; TV = television.

# Supplementary Table 8. Sources of information used not to decide to receive the HPV vaccine (multiple answers allowed)

|  | **Mothers** | | **Female adolescents** | |
| --- | --- | --- | --- | --- |
|  | **Neutral willingness* (n=800)** | **Negative willingness** (n=643)** | **Neutral willingness* (n=223)** | **Negative willingness** (n=134)** |
| TV news or health-related programs | 481 (60.13) | 498 (77.45) | 60 (26.91) | 72 (53.73) |
| Newspapers or magazines | 139 (17.38) | 168 (26.13) | 18 (8.07) | 17 (12.69) |
| Internet (news website) | 75 (9.38) | 77 (11.98) | 7 (3.14) | 7 (5.22) |
| Internet (pharmaceutical company website or health-related information site) | 10 (1.25) | 13 (2.02) | 1 (0.45) | 1 (0.75) |
| Internet (academia or hospital/clinic website) | 11 (1.38) | 14 (2.18) | 6 (2.69) | 3 (2.24) |
| Internet (MHLW website) | 18 (2.25) | 16 (2.49) | 2 (0.90) | 2 (1.49) |
| Internet (local government) | 17 (2.13) | 11 (1.71) | 2 (0.90) | 4 (2.99) |
| Internet (SNS [SM] or video-sharing site) | 22 (2.75) | 26 (4.04) | 10 (4.48) | 5 (3.73) |
| Internet (websites other than the above) | 12 (1.50) | 14 (2.18) | 1 (0.45) | 2 (1.49) |
| Doctors, staff, leaflets, or posters (academia hospital/clinic) | 81 (10.13) | 67 (10.42) | 23 (10.31) | 21 (15.67) |
| Leaflets or posters (MHLW) | 6 (0.75) | 5 (0.78) | 0 (0.00) | 2 (1.49) |
| Staff, leaflets, or posters (local government or public health center) | 53 (6.63) | 26 (4.04) | 9 (4.04) | 3 (2.24) |
| Leaflets or posters (school) | 3 (0.38) | 4 (0.62) | 0 (0.00) | 4 (2.99) |
| Lessons (school) | NA | NA | 9 (4.04) | 9 (6.72) |
| Information from acquaintances or family members | 132 (16.50) | 133 (20.68) | 46 (20.63) | 53 (39.55) |
| Public lectures or seminars by experts | 14 (1.75) | 16 (2.49) | 6 (2.69) | 3 (2.24) |
| Other than the above | 20 (2.50) | 21 (3.27) | 8 (3.59) | 4 (2.99) |
| Not applicable | 174 (21.75) | 36 (5.60) | 100 (44.84) | 31 (23.13) |

Data are reported as n (%). *As reflected by an answer of “Do not know”. **As reflected by answers of “Not willing” or “Very unwilling.”

HPV = human papillomavirus; MHLW = Ministry of Health, Labour and Welfare; NA = not applicable; SM = social media; SNS = social networking services; TV = television.

# Supplementary Table 9. Sources of information used by HCPs to decide whether to recommend the HPV vaccine (multiple answers allowed)

|  | **Recommend (n=371)** | **Positive willingness***  **(n=235)** |
| --- | --- | --- |
| TV news or health-related programs | 54 (14.56) | 47 (20.00) |
| General (non-medical) newspapers or magazines | 43 (11.59) | 34 (14.47) |
| Internet (news website) | 17 (4.58) | 14 (5.96) |
| Internet (pharmaceutical company website) | 33 (8.89) | 23 (9.79) |
| Internet (medical news sites for doctors [e.g., m3/MR-kun, So-net, etc.]) | 80 (21.56) | 57 (24.26) |
| Internet (academia or hospital/clinic website) | 60 (16.17) | 17 (7.23) |
| Internet (MHLW website) | 44 (11.86) | 17 (7.23) |
| Internet (local government) | 20 (5.39) | 6 (2.55) |
| Internet (SNS [SM] or video-sharing website) | 16 (4.31) | 3 (1.28) |
| Internet (websites other than the above) | 1 (0.27) | 2 (0.85) |
| Leaflets or posters (MHLW) | 76 (20.49) | 50 (21.28) |
| Academic conferences or lectures | 195 (52.56) | 87 (37.02) |
| Medical magazines, literature, or scientific articles | 195 (52.56) | 99 (42.13) |
| Information from pharmaceutical company staff | 54 (14.56) | 32 (13.62) |
| Information from colleagues | 60 (16.17) | 32 (13.62) |
| Information from acquaintances or family members | 6 (1.62) | 4 (1.70) |
| Other than the above | 6 (1.62) | 4 (1.70) |
| Not applicable | 20 (5.39) | 24 (10.21) |

Data are reported as n (%). *As reflected by answers of “Willing” or “Very willing.”

HCP = healthcare professional; HPV = human papillomavirus; MHLW = Ministry of Health, Labour and Welfare; NA = not applicable; SM = social media; SNS = social networking services; TV = television.

# Supplementary Table 10. Sources of information used by HCPs not to decide to recommend the HPV vaccine (multiple answers allowed)

|  | **Neutral willingness* (n=239)** | **Negative willingness****  **(n=74)** |
| --- | --- | --- |
| TV news or health-related programs | 108 (45.19) | 35 (47.30) |
| General (non-medical) newspapers or magazines | 79 (33.05) | 23 (31.08) |
| Internet (news website) | 17 (7.11) | 10 (13.51) |
| Internet (pharmaceutical company website) | 17 (7.11) | 3 (4.05) |
| Internet (medical news sites for doctors [e.g., m3/MR-kun, So-net, etc.]) | 66 (27.62) | 18 (24.32) |
| Internet (academia or hospital/clinic website) | 24 (10.04) | 5 (6.76) |
| Internet (MHLW website) | 27 (11.30) | 7 (9.46) |
| Internet (local government) | 14 (5.86) | 2 (2.70) |
| Internet (SNS [SM] or video-sharing website) | 5 (2.09) | 1 (1.35) |
| Internet (websites other than the above) | 4 (1.67) | 0 (0.00) |
| Leaflets or posters (MHLW) | 55 (23.01) | 10 (13.51) |
| Academic conferences or lectures | 46 (19.25) | 20 (27.03) |
| Medical magazines, literature, or scientific articles | 58 (24.27) | 17 (22.97) |
| Information from pharmaceutical company staff | 29 (12.13) | 4 (5.41) |
| Information from colleagues | 38 (15.90) | 13 (17.57) |
| Information from acquaintances or family members | 7 (2.93) | 6 (8.11) |
| Other than the above | 6 (2.51) | 7 (9.46) |
| Not applicable | 25 (10.46) | 3 (4.05) |

Data are reported as n (%). *As reflected by an answer of “Do not know.” **As reflected by answers of “Not willing” or “Very unwilling.”

HCP = healthcare professional; HPV = human papillomavirus; MHLW = Ministry of Health, Labour and Welfare; SM = social media; SNS = social networking services; TV = television.
